# Construction of heat stress regulation networks based on Illumina and SMRT sequencing data in potato

**DOI:** 10.3389/fpls.2023.1271084

**Published:** 2023-11-02

**Authors:** Lina Shang, Yonghong Zhou, Shiqi Wen, Ke Wang, Yang Li, Meihua Zhang, Hongju Jian, Dianqiu Lyu

**Affiliations:** ^1^ Integrative Science Center of Germplasm Creation in Western China (CHONGQING) Science City, Southwest University, Chongqing, China; ^2^ College of Agronomy and Biotechnology, Southwest University, Chongqing, China; ^3^ State Cultivation Base of Crop Stress Biology for Southern Mountainous Land of Southwest University, Southwest University, Chongqing, China; ^4^ Chongqing Key Laboratory of Biology and Genetic Breeding for Tuber and Root Crops, Southwest University, Chongqing, China

**Keywords:** potato, heat stress, NGS sequencing, SMRT sequencing, alternative splicing, lncRNAs

## Abstract

Potato (*Solanum tuberosum* L.) is one of the most important tuber food crops in the world; however, the cultivated potatoes are susceptible to high temperature, by which potato production is adversely affected. Understanding the coping mechanism of potato to heat stress is essential to secure yield and expand adaptability under environmental conditions with rising temperature. However, the lack of heat-related information has significantly limited the identification and application of core genes. To gain deeper insights into heat tolerance genes, next-generation sequencing and single-molecule real-time sequencing were used to learn the transcriptional response of potato to heat stress and 13,159 differentially expressed genes (DEGs) were identified in this study. All DEGs were grouped into 12 clusters using the K-means clustering algorithm. Gene Ontology enrichment analysis revealed that they were involved in temperature signaling, phytohormone, and protein modification. Among them, there were 950 differentially expressed transcription factors (DETFs). According to the network analysis of DETFs at the sixth hour under heat stress, we found some genes that were previously reported to be associated with photoperiodic tuberization, *StCO* (*CONSTANS*), tuber formation, *StBEL11* (*BEL1-LIKE 11*), and earliness in potato, *StCDF1* (*CYCLING DOF FACTOR 1*) responding to temperature. Furthermore, we verified the relative expression levels using quantitative real-time polymerase chain reaction, and the results were consistent with the inferences from transcriptomes. In addition, there were 22,125 alternative splicing events and 2,048 long non-coding RNAs. The database and network established in this study will extend our understanding of potato response to heat stress. It ultimately provided valuable resources for molecular analysis of heat stress response in potato and cultivation of potato varieties with heat tolerance.

## 1 Introduction

Heat stress is usually defined as the transient rise of temperature that exceeds the ambient by 10°C–15°C (Wahid et al., 2007). It is one of the biggest environmental challenges affecting human life in almost all aspects, especially in food security. Heat stress frequently occurs in these years and significantly influences crop growth, yield, and quality (Lesk et al., 2016). Furthermore, growth and developmental processes of plants are strongly sensitive to ambient temperature fluctuations. The rising global temperature is a significant effect on the plant life cycles and agricultural productivity, by reducing spikelet fertility and grain yields, delaying seed germination, declining photosynthetic efficiency, and disrupting the seasonal growth of certain species (Fitter and Fitter, 2002; Jagadish et al., 2010; Suriyasak et al., 2020; Anderson et al., 2021). An increment of every 1°C at the global scale, the yields of soybean, rice, wheat, and maize will decrease by 3.1%, 3.2%, 6.0%, and 7.4%, respectively (Zhao et al., 2017). Therefore, it is essential to learn the effect of high temperature on plant growth to ensure crop production and food security.

Potato (*Solanum tuberosum* L.) is one of the most important non-grain commodity and tuber food crops, which is rich in carbohydrates and nutrients, such as starch, essential amino acids, vitamins, and minerals. In the past decades, the production of potato steadily increased. The potato harvesting area was up to 19 million hectares, and the total yield was more than 388 million tons in 2017 (FAO, 2023). Potatoes are mostly grown in cool climates, which tuber yield is particularly vulnerable to elevated temperature (Levy and Veilleux, 2007). Compared with cool temperature, high temperature delays or inhibits tuberization. The optimum growth temperatures for the aboveground plant of potato and for tuberization and tuber growth are different; for the former, the ideal temperature is 20°C–25°C, whereas, for the latter, 15°C–20°C is preferred (Rykaczewska, 2013). Owing to the global climate warming, potential potato yield has been predicted to decrease by 9%–18% in most parts of the world (Hijmans, 2003).

The potato tubers are formed from underground swelling stolon. The key physiological processes for potato tuber initiation and growth are chiefly accompanied by synthesis of carbohydrates through photosynthesis in leaves. Then, the photosynthetic terminal product, sucrose, is transported to the stolon and converted into starch (Huner et al., 2016). However, carbon transport from leaves to tuber is sensitive to temperature in potato. High temperatures reduce the proportion of assimilated carbon that is converted to starch in the tuber (Hancock et al., 2014). The optimum temperature for potato plant photosynthesis and biomass accumulation is about 20°C. For every 5°C of increase in temperature, the photosynthetic efficiency would decrease by 25% (Timlin et al., 2006).

Heat shock proteins (HSPs)/chaperones play critical roles in multiple stress responses. HSPs functioned as buffers to restrain protein misfolding and resolve aggregation (Jacob et al., 2017). Heat stress transcription factors (HSFs) are involved in heat stress response (HSR). In elevated temperature, HSFs could trigger the accumulation of HSPs and make plants acquire thermotolerance (Pirkkala et al., 2001). In a potato genome-wide study, 27 HSFs with diverse functions were identified (Tang et al., 2016). The expression level of heat shock cognate 70 (HSc70) was increased to stabilize the yield of potato (cv. Desiree) under moderately elevated temperature, indicating that this protein can significantly alleviate adverse effects of heat stress (Trapero-Mozos et al., 2018). Under heat stress, the expression level of eukaryotic elongation factor 1A has positive correlation with the number of potato tubers and yield (Momcilovic et al., 2016). The transcriptional level of *StSP6A*, a core tuberization signal, was reduced at ambient temperatures, along with lower tuber yield (Navarro et al., 2011; Hancock et al., 2014). The expression level of *StTOC1*, encoding an evening-expressed protein, was higher in developing potato tubers at 30°C than that at 22°C (Strayer et al., 2000). Previous report has shown that *StTOC1* affects potato tuberization by suppressing *StSP6A* autoactivation at warm temperatures (Morris et al., 2019). However, due to the limited understanding of potato heat stress tolerance at physiological, biochemical, and molecular levels, more efforts should be made to further improve their thermotolerance.

Since the draft genome of RH89-039-16 (heterozygous diploid) was released by the Potato Genome Sequence Consortium in 2011, more and more genes’ functions are being explored (Xu et al., 2011). However, there are still many functional transcripts that have not been identified. The next-generation sequencing (NGS) technology makes the remarkable success in discovering differentially expressed genes (DEGs) in potato in previous study (Moon et al., 2018; Tang et al., 2020). However, NGS was unable to obtain full-length transcripts due to read length limitations, which hinders the whole genome assembly and individual gene isolation (Giani et al., 2020). As one of the third-generation sequencing technologies, single-molecule real-time (SMRT) sequencing is sufficient to generate full-length transcripts for whole-transcriptome profiling and has distinguished progress in the study of plants such as populus and maize (Wang et al., 2016; Chao et al., 2019). Compared with the NGS, the SMRT sequencing could provide better sequence integrity of cDNA molecules from 5′ to 3′ ends and higher accuracy of identified alternative isoforms (Sharon et al., 2013). However, the SMRT sequencing is currently not able to directly quantify gene expression. In order to integrate their advantages, high-quality short reads of NGS are used to correct erroneous long reads of SMRT (Hackl et al., 2014). Therefore, the combined analysis of NGS and SMRT sequencing has been widely utilized.

In present study, NGS and SMRT techniques were used for the first time to sequence RNA of potato leaves, which were subjected to elevated temperature at 35°C for 0h–48h. Then potential heat tolerance genes, transcription factors (TFs), long non-coding RNA (lncRNA) and alternative splicing (AS) events were further identified. Elucidating the underlying mechanism could help improve the thermotolerance of potatoes and facilitate the breeding of new potato varieties, thereby increasing the yield and quality of potatoes in response to climate change.

## 2 Materials and methods

### 2.1 Plant materials, growth conditions, and heat stress treatments

“Kexin18”, a tetraploid potato cultivar, was used in this study. Plants were propagated *in vitro* on Murashige and Skoog (MS) medium supplemented with 8 g/L carrageenan and 40 g/L sucrose under long-day photoperiod (16h light/8h dark) at 22°C. Three-week-old potato plants were transferred to water for 2 weeks under the same conditions. Then, they were transferred and grown under long-day photoperiod with a light/dark temperature of 35°C (**Supplementary Figure S1**). The potato leaves were collected at 0h, 0.5h, 1h, 3h, 6h, 12h, 24h and 48h after heat stress and were immediately frozen in liquid nitrogen and stored at −80°C until analysis. Three biological replicates were collected for each treatment.

### 2.2 Construction of cDNA library for Illumina and SMRT sequencing

Total RNA was extracted each sample using EZ-10 DNA away RNA Mini-Preps Kit (Sangon Biotech, Shanghai, China) following the manufacturer’s instructions. After the quality and purity were confirmed by NanoDrop spectrophotometer (Thermo Fisher Scientific, USA), the high-quality RNAs were used for cDNA library construction. For Illumina sequencing, 24 libraries were constructed using the NEBNext Ultra RNA Library Prep Kit (NEB, USA) and sequenced on the Illumina Hiseq2500 platform in Biomarker Technologies Corporation (Beijing, China). For SMRT sequencing, the RNAs from each sample were further mixed in equal quantities. Full-length cDNA was synthesized using the SMARTer PCR cDNA Synthesis Kit (Clontech, Mountain View, CA, USA) and sequenced by Iso-Seq with PacBio RS II systems (Pacific Biosciences, Menlo Park, CA, USA).

### 2.3 Analysis of the Illumina and SMRT data

Quality-checked analysis was performed on the raw Illumina sequencing reads using FastQC tool (https://www.bioinformatics.babraham.ac.uk/projects/fastqc/) and the clean data were obtained after removing the adaptor sequences and low-quality reads using Trimmomatic (v0.35).The high-quality clean reads were then mapped to the reference genome database (http://solanaceae.plantbiology.msu.edu/rh_potato_download.shtml) using HISAT2 version 2.1.0 with the default para-meters. The counts of the mapped clean reads per gene were calculated using the FeatureCounts, then they were normalized into fragments per kilobase of tr-anscript per million mapped reads (FPKM) values using the Cufflinks software.

Raw data acquired from SMRT sequencing were processed to obtain the circular consensus sequences (CCSs) by removing polymerase reads, which were less than 50 bp and quality value lower than 0.75. The CCSs were processed into error-corrected reads of inserts (ROIs) with minFullPass ≥ 3 and minPredictedAccuracy > 0.9. The ROIs with the 5′ and 3′ cDNA primers and a poly (A) tail were considered to be full-length non-chimeric (FLNC) transcripts. All FLNC transcripts were aligned to the RH89-039-16 reference genome (http://solanaceae.plantbiology.msu.edu/rh_potato_download.shtml) using GMAP software in this study. These FLNC CCS reads were polished with the Quiver program. Finally, high-quality full-length transcripts with post-correction accuracy more than 99% were retained for further analysis. Based on the OrthoDB database (https://www.orthodb.org/), the Benchmarking Universal Single-Copy Orthologs (BUSCO) assessment (version 3.0.2) was used to evaluate the integrity of the full-length transcripts without redundancy. All raw data were deposited in the National Center for Biotechnology Information (NCBI) sequence read archive with submission ID (SUB13775583).

### 2.4 Differential expression analysis

The DEGs were screened using DESeq2 package, regarding the genes with false discovery rate (FDR) < 0.05 and the absolute fold change ≥ 2. The TF in potato genome were identified by using iTAK software, and the differentially expressed transcription factors (DETFs) were screened from the DEGs database. The DEGs involved in plant hormone metabolism or signal transduction were screened based on Gene Ontology (GO) functional annotation.

### 2.5 Weighted gene co-expression network analysis, functional annotation, and enrichment analysis of DEGs

Weighted gene co-expression network analysis (WGCNA) was constructed by R package (version 4.0.2) with FPKM values > 1. The parameters of power, minimal gene module size and threshold of merge similar modules depend on the situation. Clusters of Orthologous Groups, Pfam (protein family), Swiss-prot (a manually annotated, non-redundant database), and NR (NCBI non-redundant proteins) were used as universal tools to analysis the functional annotations of the DEGs. GO term enrichment of the DEGs was displayed with the online tool (http://www.geneontology.org/).

### 2.6 Identification of long non-coding RNAs and alternative splicing events

To identify putative lncRNAs, four computational methods, including CPC (coding potential calculator), CNCI (coding-non-coding index), Pfam protein domain analysis and CPAT (coding potential assessment tool) were used. The candidate lncRNAs were screened using the parameters with lengths longer than 200 nt and more than two exons.

AS events were identified using AStalavista tool and five AS types, including retented intron (RI), skipped exon, alternative 3′ splice site (A3), alternative 5’ splice site (A5), and mutually exclusive exons (MX) were detected based on the Illumina and SMRT data.

### 2.7 Hub DETF screening

Network visualization of DETFs with FDR < 0.01 was carried out using Cytoscape with default parameters. The maximal clique centrality (MCC) was utilized as the most effective algorithms in the CytoHubba plugin of Cytoscape software to identify hub TFs. Target genes of upregulated and downregulated TFs were searched to identify the relevant pathways and functions of DETFs. The size of dots and the number of lines represent the quantity of target genes. The more target genes that TFs have indicates that these TFs may be potential core.

### 2.8 Quantitative real-time PCR

In order to verify the accuracy of Illumina and SMRT data, quantitative real-time polymerase chain reaction (qRT-PCR) was used to detect DETFs and differentially expressed lncRNAs (DELs) identified from our RNA-seq analysis, and *StEF-1α* was used as an internal reference gene for data normalization. BIO-RAD iTaq Universal SYBR Green Supermix was used for qRT-PCR reactions on a CFX96 Real-time System (BIO-RAD, USA) with the following thermal cycling program: 95°C for 30 s, followed by 39 cycles of 95°C for 5 s and at 60°C for 30 s, then 95°C for 5 s and 60°C for 1 min. The melting curves were analyzed from 60°C to 95°C with an increment of 0.5°C/s. Three independent biological replicates for each treatment and three technical replicates for each biological replicate were performed. The method of 2^–ΔΔCt^ was used to normalize the relative expression levels of DETFs and DELs. All the primers used in the study are listed in **Supplementary Table S1**.

## 3 Result

### 3.1 Illumina- and SMRT-based RNA sequencing

**Figure 1:**
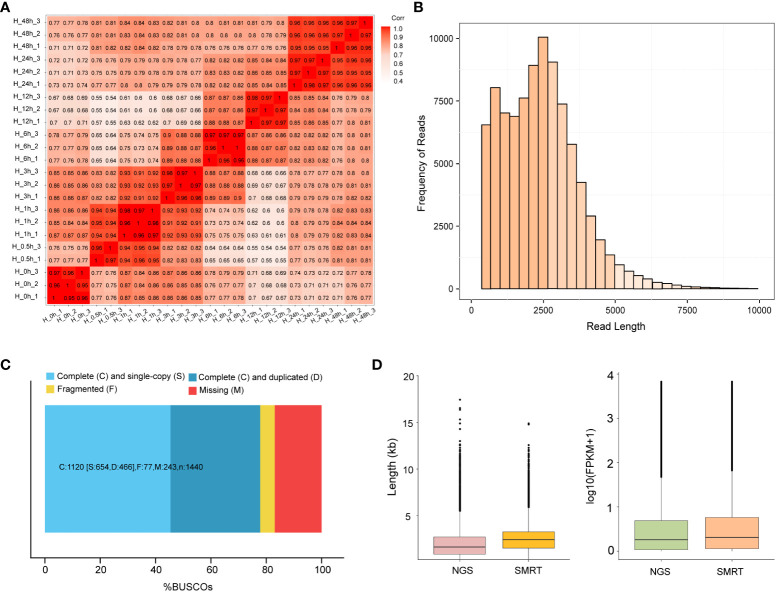
RNA-sequencing analysis based on Illumina and SMRT. **(A)** Pearson’s correlation coefficient analyses among 23 samples. H-0h represents control samples; H-0.5h represents heat stress for 30 min; H-1h represents heat stress for 1h; H-3h represents heat stress for 3h; H-6h represents heat stress for 6h; H-12h represents heat stress for 12h; H-24h represents heat stress for 24h; H-48h represents heat stress for 48h. Except for H-0.5h, which had two biological replicates, all the others had three biological replicates. **(B)** The distributions of the consensus isoforms read numbers and lengths from PacBio libraries. The *x*-axis represents the consensus isoforms read length; the *y*-axis represents the number of consensus isoforms. **(C)** BUSCO completeness assessments for genomics data quality control. Bar charts produced with the BUSCO plotting tool show proportions classified as complete (C, blues), complete single-copy (S, light blue), complete duplicated (D, dark blue), fragmented (F, yellow), and missing (M, red). **(D)** The differences in length and FPKM of NGS and SMRT sequencing. The left figure represents the difference in sequencing length between NGS and SMRT. The right figure represents the FPKM differences between NGS and SMRT sequencing.

To analyze the heat stress regulation network of potato, 24 cDNA libraries at different time points were sequenced on the Illumina Hi-Seq platform. An average of 48.05 M reads was obtained for each sample. After the removal of adaptor and low-quality reads, 43.50 M clean reads were obtained and subsequently mapped to the potato reference genome RH89-039-16, and 69.77% of the reads were uniquely mapped. The Q30 values of 24 samples were higher than 93.27%, indicating that the sequencing data were high quality and were suitable for subsequent analysis (**Supplementary Table S2**). Principal Component Analysis (PCA) was performed between the three samples at each time points, and showed that H-0.5 h-2 sample was less consistent with the other two replicates, while the rest samples had a high consistency (**Supplementary Figure S2**). Pearson’s correlation coefficient analyses of all samples verified the consistency of biological replicates collected at each time point after removing H-0.5 h-2 sample (**Figure 1A**).

To obtain a representative full-length transcriptome for potato, the total RNA from 8 time points were mixed with equal quantity and used for sequencing by SMRT technology. In this study, a total of 21.38 Gb clean data was generated and 289,281 CCSs were extracted from the raw data. Among CCSs, there were 245,298 FLNC reads and 31,535 undesired primer reads. According to the clustering algorithm, 94,500 consensus isoform sequences were identified, including 89,716 high-quality isoforms and 4,379 low-quality isoforms (**Table 1**). After error correction, redundant transcripts were removed. Then, we obtained 58,747 high-quality full-length transcript sequences with an average length of 2,340 bp, which are longer than Illumina sequencing data. The length distribution of consensus isoform reads is shown in **Figure 1B**. In addition, the integrity of the de-redundant transcriptome was assessed by BUSCO, and 77.8% of the isoforms were intact (**Figure 1C**). These results confirmed the reliability of full-length transcriptome for following analysis. Comparing to the Illumina platform producing short reads, the PacBio SMRT platform provided long reads, making it possible to accurately reconstruct full-length splice variants (**Figure 1D**).

**Table 1:** SMRT sequencing output statistics in this study.

SMRT	Number
Data size (G)	21.38
Number of CCS	289,281
Read bases of CCS	650,042,591
Mean read length of CCS	2,247
Mean number of passes	38
Number of undesired primer reads	31,535
Number of filtered short reads	55
Number of full-length non-chimeric reads	245,298
Full-length non-chimeric percentage (FLNC%)	84.80%
Number of consensus isoforms	94,500
Average consensus isoforms read length	2,340
Number of polished high-quality isoforms	89,716
Number of polished low-quality isoforms	4,379

### 3.2 Screening and functional enrichment of DEGs in response to heat stress

**Figure 2:**
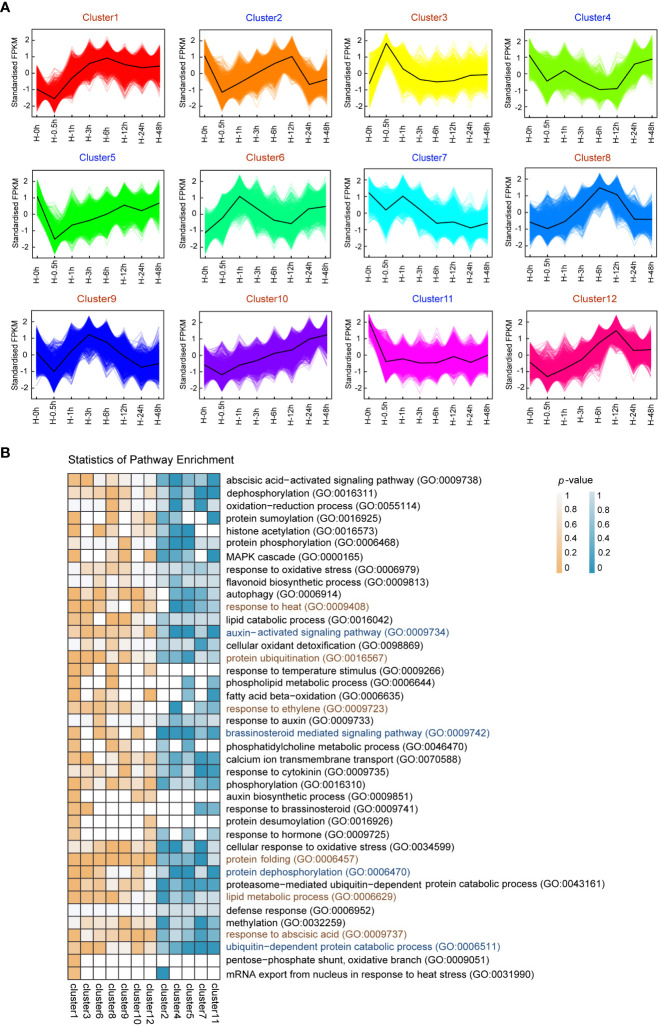
K-means clustering and enrichment analysis of differentially expressed genes (DEGs) among 23 samples. **(A)** K-means clustering showing the transcriptome expression profiles. 12 clusters were identified based on expression levels in eight samples (0h, 0.5h, 1h, 3h, 6h, 12h, 24h and 48h after heat stress). **(B)** Gene Ontology (GO) enrichment among 12 clusters. The color bar indicates enrichment degrees from low (white) to high (orange or indigo).

In order to investigate gene expression changes in response to heat stress in potato leaves, a total of 13,159 DEGs were screened in 7 time points, including small HSPs (**Supplementary Figure S3**, **Supplementary Table S3**). The number of DEGs at each time point of treatment is diverse, with the minimum at the 0.5th hour (with 783 upregulated and 725 downregulated genes) and the maximum at the sixth hour (with 3,937 up and 3,874 downregulated) (**Supplementary Figure S4**), indicating that DEGs play central roles in confronting adversity at the sixth hour after heat stress. Using the K-means clustering algorithm, all DEGs were grouped into 12 clusters (clusters 1–12) (**Figure 2A**). DEGs from the clusters 3 and 6 were upregulated at the 0.5th and first hour after heat stress, respectively. In contrast, DEGs from the cluster 5 were downregulated at the 0.5th hour after heat stress and increased gradually, while the expression levels of DEGs from the cluster 11 decreased dramatically from the start and kept a steady state after 0.5h. These results suggested that these DEGs were potentially involved in response to heat stress at the early stage. DEGs from the clusters 8 and 12 were predominantly expressed at the sixth and 12th hour after heat stress, respectively, which indicated that these DEGs played crucial roles in response to heat stress in the intermediate period.

To better understand the functions of DEGs, GO enrichment analysis were performed (**Figure 2B**). The early upregulated genes under heat stress in clusters 3 and 6 were mainly enriched in response to heat (GO:0009408), protein ubiquitination (GO:0016567), and lipid metabolic process (GO:0006629). The early downregulated genes in clusters 5 and 11 were mainly enriched in auxin-activated signaling pathway (GO:0009734), brassinosteroid mediated signaling pathway (GO:0009742), and protein dephosphorylation (GO:0006470). These results indicated that the DEGs that respond to early heat stress were mainly involved in temperature signal, phytohormone and protein modification in potato. In clusters 8, 9, and 12, upregulated genes during intermediate heat stress were mainly enriched protein folding (GO:0006457) and response to abscisic acid (GO:0009737).

**Figure 3:**
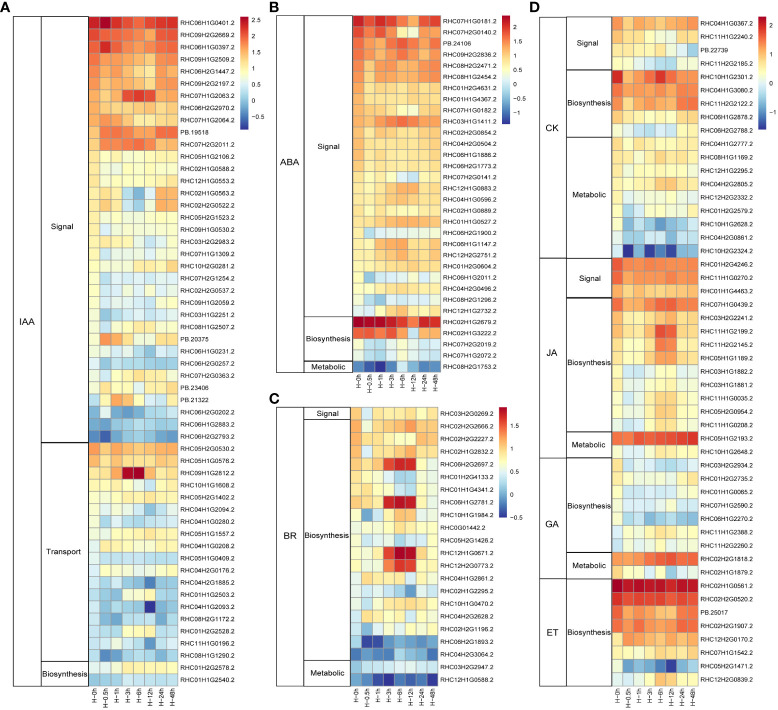
Heat maps of the differentially expressed phytohormones in potato under heat stress. **(A)** DEGs in auxin (IAA) signal, transduction and biosynthesis pathways. **(B)** DEGs in abscisic acid (ABA) signal, biosynthesis, and metabolic pathways. **(C)** DEGs in brassinosteroid (BR) signal, biosynthesis, and metabolic pathways. **(D)** DEGs associated with signal, biosynthesis, and metabolic pathways of these genes including cytokinin (CK), jasmonic acid (JA), gibberellin (GA), and ethylene (ET) after heat stress treatment.

Phytohormones are crucial signal compounds, which could regulate plant growth, development, and environmental stress responses (Waadt et al., 2022). To understand the processes of phytohormone signal under heat stress in potato, DEGs involved in phytohormone perception, biosynthesis, transport, signal transduction, and degradation processes were selected (**Figure 3**). Among them, 55 DEGs (35 genes in signal transduction, 18 genes in auxin (IAA) transport, and two synthetic pathway genes) were involved in IAA pathway (**Figure 3A**). As stress hormone, ABA plays critical roles in response to abiotic stress in plants. In this study, ranking second only to IAA, 32 DEGs involved in ABA pathways were detected. Similar with IAA pathway, more than 84.3% (27 of 32) DEGs were involved in the signal transduction pathway (**Figure 3B**). As the homologous gene of *RHC03H1G1411.2*, *AtPYL8* plays an important role for ABA signaling and drought stress responses in *Arabidopsis* (Lim et al., 2013). CK was mainly involved in metabolic pathways in potato leaves to response heat stress, while the synthetic pathway genes dominated in the hormones of BR, JA, GA, and ET with 10, 19, 7, and 8 genes, respectively (**Figures 3C, D**). Taken together, potato respond to heat stress mainly through signal transduction pathways in both IAA and ABA, while the contents of BR, CK, JA, GA, and ET were changed through synthetic and metabolic pathways.

### 3.3 Construction and validation of DETF networks in response to heat stress

**Figure 4:**
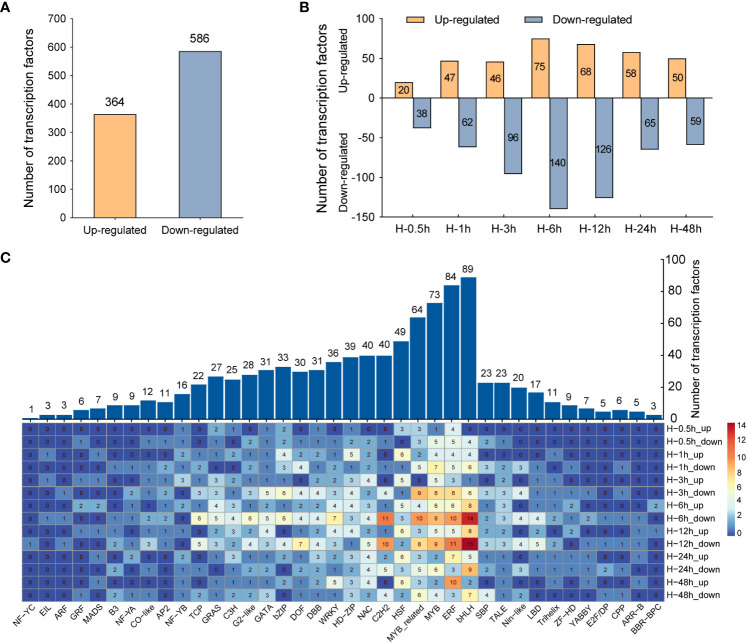
Comparative analysis of differentially expressed transcription factors (DETFs). **(A)** The total number of upregulated and downregulated transcription factors under heat stress. **(B)** The quantitative distribution of upregulated and downregulated transcription factors at different time points after heat stress. **(C)** Distribution of DETFs families. The numbers indicate the number of DETFs in different families.

TFs, as major regulators, can regulate the expression of stress-related genes and play critical roles in enhancing plant tolerance to abiotic stresses, such as heat, drought, and cold. In the genome, approximately 10% of genes codes for TFs (Brivanlou and Darnell, 2002). Therefore, DETFs were screened and further comprehensively analyze in this study. A total of 950 DETFs were detected, of which 364 TFs were upregulated and 586 TFs were downregulated (**Figure 4A**). The number of TFs downregulated was far more than that upregulated in the samples after heat stress. At the sixth hour after heat stress, the number of downregulated genes is almost twice that of upregulated genes (**Figure 4B**). Furthermore, family analysis of DETFs showed that these TFs were classified into 38 families, among which the bHLH family had the largest number of DETFs (89), followed by ERF (84), MYB (73), MYB related (64), and HSF (49), respectively. In the above TF families, the number of downregulated TFs was significantly greater than that of upregulated TFs in bHLH, ERF, MYB, and MYB-related families, while more TFs were upregulated rather than downregulated in HSF families (**Figure 4C**), which was consistent with the commonly induced expression of HSFs in heat stress (Liu et al., 2011).

**Figure 5:**
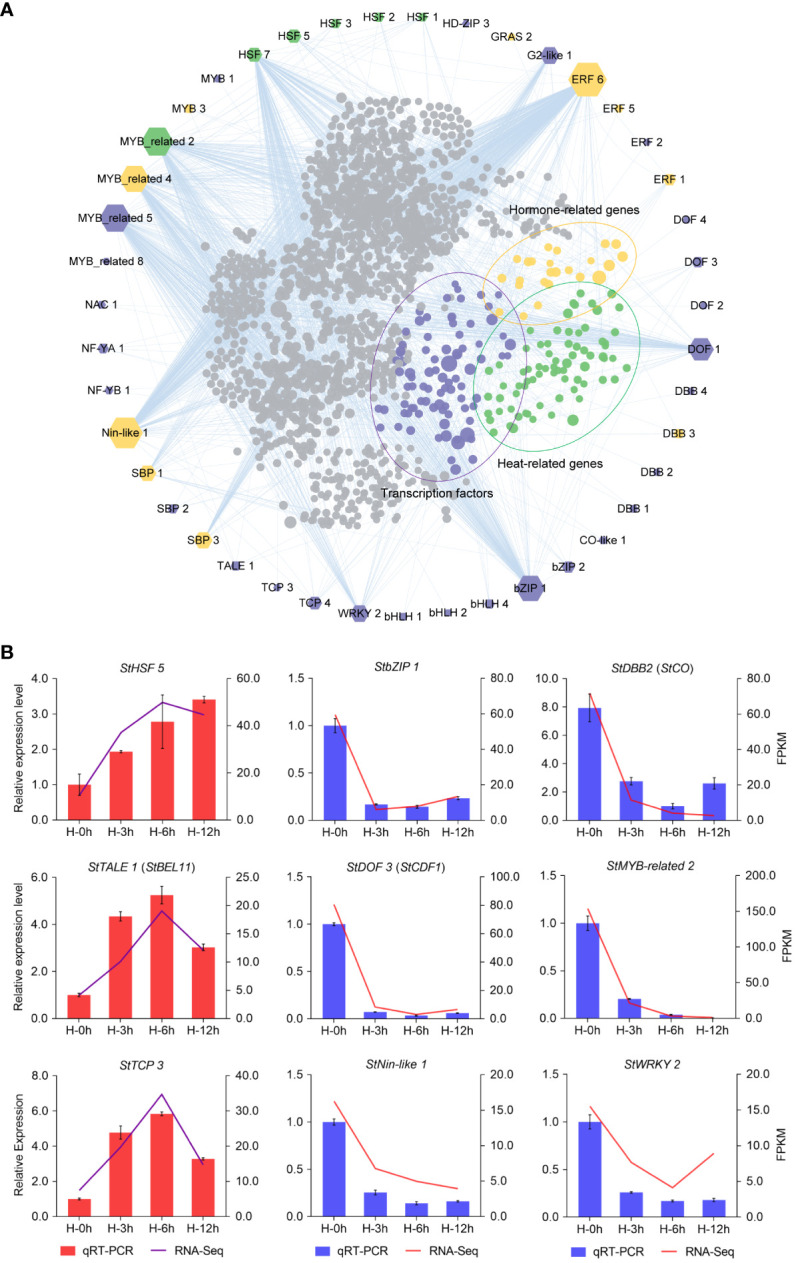
Network analysis and quantitative real-time PCR (qRT -PCR) validation of differentially expressed transcription factors (DETFs) at the sixth hour after heat stress. **(A)** The edges are the mainly DETFs at the sixth hour after heat stress, and the inners are the target genes corresponding to the DETFs. Target genes (inners) of transcription factors (edges) are indicated by purple, yellow, and green dots, which represent transcription factors, hormone-related genes, and heat-related genes, respectively. The size of the polygon indicates the number of target genes from less (small) to more (big). **(B)** qRT-PCR validation of DETFs.

As shown in **Figure 4B**, the number of DETFs at the sixth hour under heat stress was the maximum. Therefore, we analyzed and visualized the core DETFs and their target genes by PlantTFDB data. Then, a putative functional network was set up (**Figure 5**). Among them, there were more target genes related to *bZIP1*, *DOF1*, *ERF6*, *HSF7*, *MYB-related2/4/5*, and *Nin-like1*, and many of these target genes were TFs, heat-related, and hormone-related genes (**Figure 5A**). It was suggested that these core DETFs may played an important role in potato heat stress. Furthermore, nine DETFs were randomly selected for qRT-PCR, including the gene for photoperiodic tuberization, *StCO* (*StDBB2*) (Gonzalez-Schain et al., 2012), tuber formation, *StBEL11* (*StTALE1*) (Ghate et al., 2017), and earliness in potato, *StCDF1* (*StDOF3*) (Kloosterman et al., 2013), which verified the perfect RNA-seq data (**Figure 5B**).

### 3.4 Identification of long non-coding RNA

**Figure 6:**
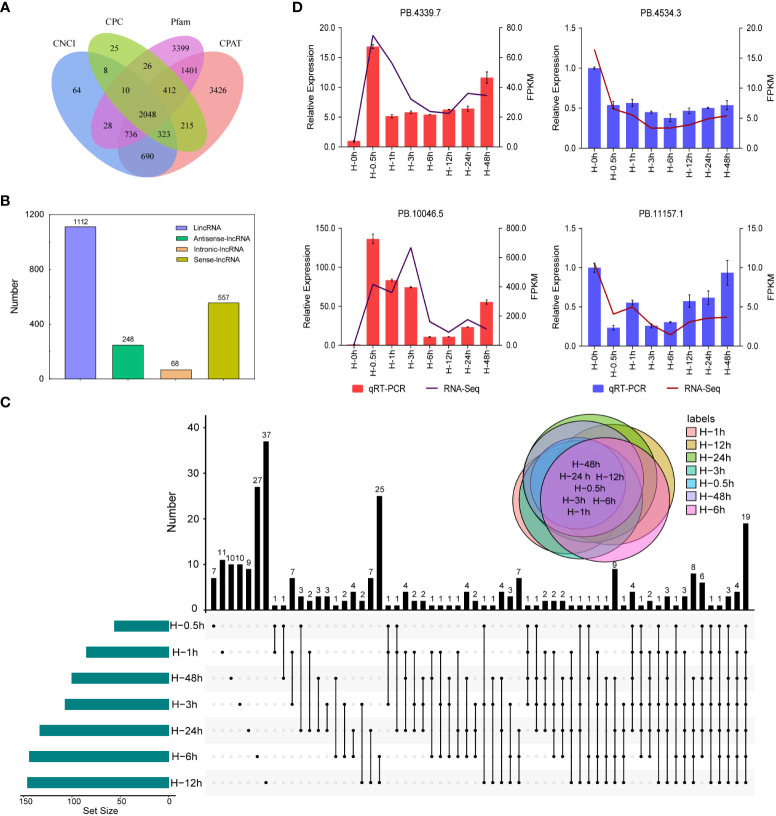
Identification and quantitative real-time PCR (qRT -PCR) validation of long non-coding RNAs (lncRNAs). **(A)** Venn diagram of the number of lncRNAs predicted by Calculator (CPC), Coding-Non-Coding Index (CNCI), Coding-Potential Assessment Tool (CPAT), and pfam protein structure domain analysis. **(B)** Distribution of different lncRNAs. **(C)** Venn diagram of differentially expressed lncRNAs detected in seven samples. **(D)** qRT-PCR validation of differentially expressed lncRNAs.

Plant lncRNAs are the major component in transcriptome sequencing, which could regulate transcription through chromatin modifications and alter the behavior of target proteins (Yu et al., 2019). However, little information about lncRNA has been obtained in potato. In this study, a total of 2,048 putative lncRNAs were predicted using the SMRT sequencing technology (**Figure 6A**). These lncRNAs were mapped on the reference genome and classified to sense, antisense, intergenic, and intronic lncRNAs. Among them, more than 56.02% (1,112) of lncRNAs were located in the intergenic region while less than 3.43% (68) were located in the intronic region (**Figure 6B**). To identify the key lncRNAs related to heat stress, we obtained 262 DELs in the library of seven samples heat treated at different times, including common and unique genes. The number of DELs at the 12th hour after heat stress was the maximum, with a total of 37 (**Figure 6C**). Furthermore, four lncRNAs, namely, PB.4339.7, PB.10046.5, PB.4534.3, and PB.11157.1, were randomly selected for qRT-PCR verification, which were highly consistent with the RNA-seq results (**Figure 6D**).

### 3.5 Analysis of alternative splicing

**Figure 7:**
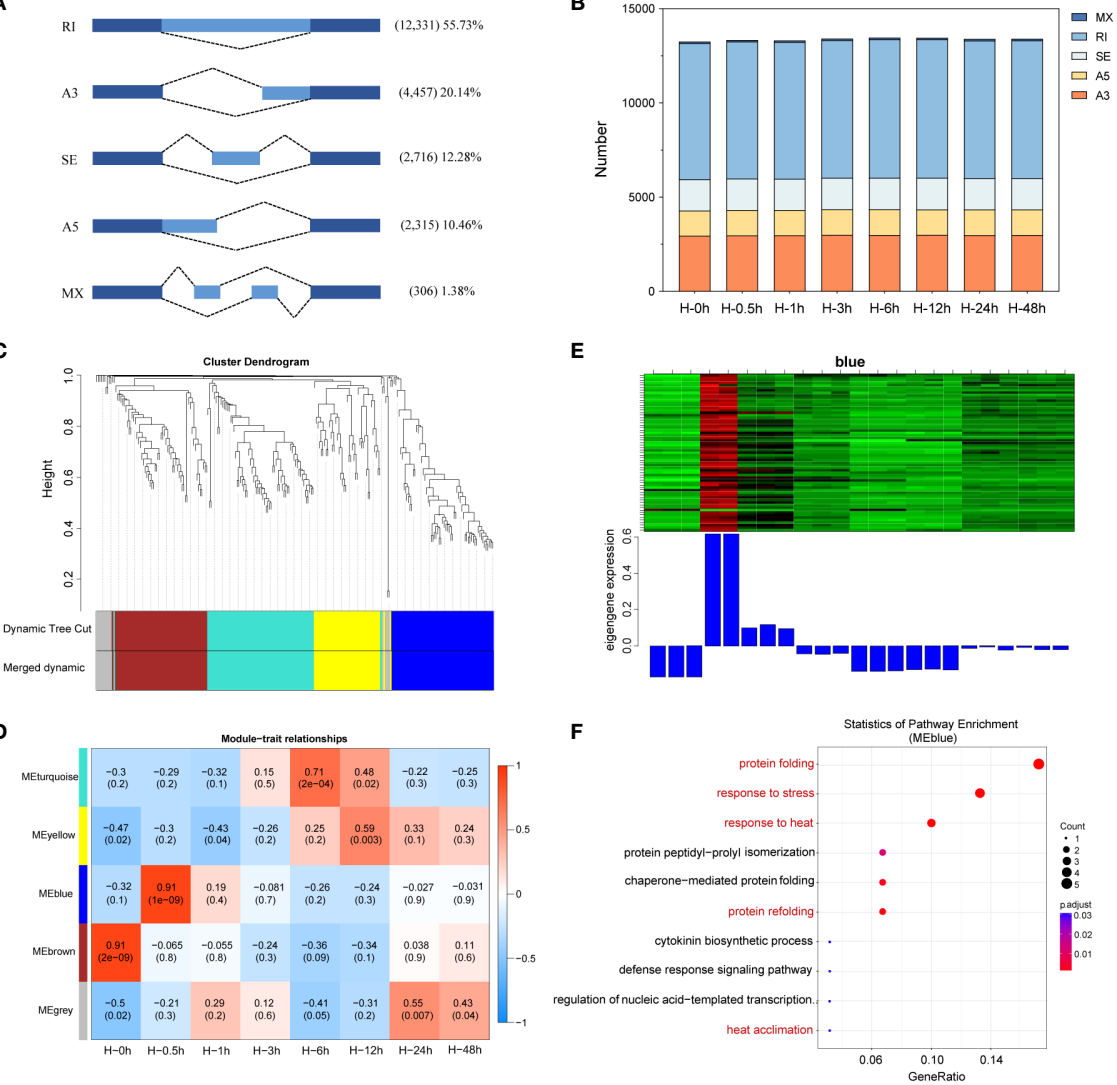
Identification of alternative splicing (AS) events and enrichment analysis of differentially alternatively spliced (DAS) genes. **(A)** Classification of AS events identified. RI, intron retention; A3, alternative 3′ splice site; SE, exon skipping; A5, alternative 5′ splice site; MX, mutually exclusive exon. **(B)** The number of AS events in eight time points. **(C)** Weighted gene co-expression network (WGCNA) analysis of gene clustering number and modular cutting. Each branch represents a gene and each color below represents a gene co-expression module. Dynamic tree cut indicates the modules divided based on the gene clustering results. Merged dynamic indicates the modules divided by combining modules with similar expression patterns. **(D)** Association analysis of gene co-expression network modules with the time of heat stress. The horizontal axis represents different time point, and the vertical axis represents the eigenvectors of each module. The red lattice represents a positive correlation between the heat stress time with the module, while the blue lattice represents a negative correlation. **(E)** Expression levels of all genes and corresponding ME in blue modules. The above figure (heat map) shows the expression level of all genes in blue modules. The row represents all genes in the module, and the column represents each sample. The below figure shows the ME expression level of the module in the sample. **(F)** Gene Ontology (GO) enrichment of AS events involved in blue module.

AS can increase genetic diversity in plants, involving in biological processes from plant growth and development to stress responses (James et al., 2012). Although complex AS patterns have been revealed by genome-wide studies in plants, whether AS patterns impact the heat stress defense of potatoes is not known. In this study, a total of 22,125 AS events were detected and categorized into five AS types, including RI, SE, A3, A5, and MX by SMRT sequencing. RI was the most frequent type of AS detected, accounted for 55.73%, followed by A3, SE, A5, and MX (**Figure 7A**). Further subdivided at each time point, there was similar number of AS events among heat stressed potato leaves (**Figure 7B**), which was consistent with previous studies in potato involved in response to drought stress (Jian et al., 2022).

To establish the co-expression and correlation networks of differentially alternatively spliced (DAS) genes, WGCNA was conducted. The significantly correlated blue module was identified at the 0.5th hour sample after heat stress (*r* = 0.91, *P* = e^-9^) (**Figures 7C–E**). To learn the functions of these genes in blue module, GO enrichment analysis was performed. The results showed that these genes were mainly enriched in protein folding, response to stress and heat, chaperone-mediated protein folding, and heat acclimation (**Figure 7F**).

## 4 Discussion

Potato plays critical roles in ensuring global food security, reducing human starvation, and improving nutrition. However, potato growth and productivity were negatively affected by many environmental factors, of which heat stress has become one of the most important and uncontrollable factors (Campbell et al., 2021). Previous researches have focused on physiological and growth responses of potato cultivars to heat stress (Tang et al., 2018). The heat tolerance mechanism of potato, which is fundamental for stress resistance breeding, is still unclear. Over the past few years, RNA-seq technology using the Illumina platform has been widely utilized for quantifying gene expression due to the high-quality reads and the low costs. However, the limitations of short-read sequencing make it impossible to accurately obtain assembly of complete isoform structures. By contrast, SMRT sequencing technology using PacBio system overcomes these limitations by generating kilobase-sized sequence reads, which represent full-length mRNA molecules (Eid et al., 2009; Sharon et al., 2013). In this study, DEG, DETF, DEL, and DAS events in response to heat stress in potato were identified based on the combination of Illumina and SMRT RNA-seq data.

### 4.1 DEGs and DETFs involved in heat stress response

RNA-seq methods have been applied to identify gene networks in many crops. Heat stress can lead to differential expression of many genes. In our study, we treated potato leaves at 35°C and obtained 13,159 DEGs, of which the number of DEGs, whether upregulated or downregulated, reached the maximum at the sixth hour under heat stress, with 3,937 and 3,874 DEGs, respectively. Furthermore, K-mean analysis unveiled that the genes in cluster 8 were upregulated under heat stress for 6h. GO analysis revealed that these genes were related to oxidation-reduction, lipid catabolism, and protein folding process. In white clover, increased antioxidant defense system enabled to reduce heat-induced oxidative damage in leaves (Liu et al., 2021). Heat stress also led to the misfolding and denaturation of proteins. Therefore, the activation of above stress response genes indicated the resistance of potato to heat stress. In addition, many DEGs are involved in hormone regulation. Phytohormones regulate multiple cellular processes in plant responses to different adverse environmental conditions (Wani et al., 2016). Auxins, CK, GA, and BR are traditionally associated with plant development, while ABA is considered to be involved in abiotic stress response and tolerance of plants (Ciura and Kruk, 2018). In this study, 55 DEGs involved in IAA pathways were detected and more than 63.6% (35 of 55) DEGs function in the signal transduction pathway. Among them, RHC03H1G2251.2 encodes the IAA8 protein. In Arabidopsis, AtIAA8 interacted with the TIR1 auxin receptor and ARF TFs, involved in lateral root formation (Arase et al., 2012). The TF MdIAA27, the homologous gene of *RHC12H1G0553.2*, positively regulates phosphate uptake by promoting adventitious root development in apple (Zhao et al., 2022). It is speculated that heat stress may regulate root development through IAA signal transduction pathway and then affect plant growth. There were 32 DEGs in ABA pathway, second only to IAA. Most of these genes are involved in signal transduction, such as *StPYL4-like* (RHC06H1G1147.2), *StPYL8-like* (RHC03H1G1411.2), and *StPYL9-like* (RHC08H2G2471.2). In wheat, ABA receptor gene *TaPYL1-1B* increased the water-use efficiency, promoting drought tolerance and grain yield (Mao et al., 2022). In Arabidopsis, overexpression of *ZmPYL8*, *ZmPYL9*, and *ZmPYL12* genes can increase the resistance to drought (He et al., 2018). Therefore, it was speculated that ABA signal transduction pathway also played an important role in potato response to heat stress.

TFs are involved in a variety of plant physiological processes, which could be activated by different signals and participate in the expression regulation of stress-responsive genes. A previous study in *Brassica rapa* showed that large number of TFs were involved in response to heat stress (Dong et al., 2015). Our investigation in the heat stressed potato leaves revealed that there was a total of 950 DETFs (364 upregulated and 586 downregulated), belonging to 38 different families. At the sixth hour after heat stress, the number of DETFs was the highest, with 215, including bHLH, ERF, MYB, HSF, and DOF families. The bHLH TFs respond to drought, salt, and cold stress in plants (Sun et al., 2018). The ERF family TFs were positioned as candidate factors for studying the interaction between abiotic stress and hormones (Mizoi et al., 2012). MYB protein functions were demonstrated from different aspects, including drought stress, salt, and temperature tolerance (Wang et al., 2021). However, the knowledge of the mechanisms in potato response to heat stress is still limited. MYB TFs are one of the largest protein families. In potato, 158 *StMYB* genes were identified (Sun et al., 2019). In transcriptome data, we identified that the expression of *StMYB-related 2* was significantly downregulated at the sixth hour after heat stress. The network showed that many of its target genes were related to heat. Furthermore, qRT-PCR analysis validated the RNA-seq results. As its homologous gene, RVE8/LCL5 binds to the promoter of key clock component *TOC1* (Timing of CAB expression 1) and regulates its circadian expression in *Arabidopsis* (Farinas and Mas, 2011). In potato, *StTOC1* links environmental signaling with potato tuberization by suppressing *StSP6A* (Flowering Locus T ortholog) autoactivation in the stolons (Morris et al., 2019). Therefore, it is speculated that *StMYB-related 2* regulates the expression of *StTOC1*, which in turn affects the expression of *StSP6A* under heat stress. HSFs are the important components of a signal transduction chain, which mediate the activation of heat responsive genes. The HRS is regulated by HSFs, which are highly conserved in eukaryotes. Among the plant HSFs, *HsfA1*, considered as the master transcriptional activator, play a unique role and is indispensable for arousing the HSR (Yoshida et al., 2011). In our data, *StHSF5* was positively regulated under heat stress, which is similar to the expression of HSFs previously reported in Arabidopsis. Therefore, it was speculated that *StHSF5* plays a positive role in potato under heat stress. *SlCDFs*, members of DOF family, are involved in abiotic stress tolerance and flowering time in tomato (Corrales et al., 2014). *StCDF1* has been shown to induce tuberization by inhibiting the levels of *StCO1/2*, which significantly increased the expression levels of *StSP6A* (Navarro et al., 2011; Gonzalez-Schain et al., 2012; Kloosterman et al., 2013). In our study, *StCDF1* (*StDOF3*) and *StCO* (*StDBB2*) were downregulated under heat stress, shown in both RNA-seq and qRT-PCR. High temperature is a negative regulator of tuberization. Therefore, it is speculated that heat stress may reduce tuberization by repressing the expression level of *StCDF1*. In addition, *StCO* could be regulated by other TFs in addition to *StCDF1*.

### 4.2 lncRNAs involved in heat stress response

Recently, lncRNAs are reported as key regulators of gene expression in plant development and response to environmental stimuli. In Arabidopsis, *COLDAIR* (COLD INDUCED LONG ANTISENSE INTRAGENIC RNA) participated in the repression of *FLC* after vernalization (Swiezewski et al., 2009). *StCDF1*, together with a lncRNA counterpart, named *StFLORE*, was involved in drought stress responses in potato (Gonzales et al., 2021). However, there are few reports about systematic identification of lncRNAs under heat stress in potato. In this study, among the 2,048 putative lncRNAs, 262 lncRNAs were differentially expressed under heat stress. Further verification revealed that four randomly selected lncRNAs were differentially expressed under high-temperature signaling. Based on genomic location, lncRNAs can be categorized as sense lncRNAs, antisense lncRNAs (transcribed in an antisense orientation from genes coding for proteins), intronic lncRNAs, and intergenic lncRNAs. PB.10046.5 was transcribed from the RHC06H2G2958.2 gene encoding a NudC domain-containing protein. In Arabidopsis, overexpression of the NMig1 gene encoding a NudC domain protein was associated with strong upregulation of genes encoding HSPs and abiotic stress-associated genes (Velinov et al., 2020). PB.4339.7, PB.4534.3 and PB.11157.1 belong to the intergenic lncRNAs. Long-intergenic noncoding RNAs (lincRNAs) are located outside protein-coding genes. Plant lincRNAs are functionally diverse lncRNAs and have been the focus of lncRNA research. With the advent of high throughput technologies, several lincRNAs were identified in plant. For example, *DRIR* (*DROUGHT INDUCED LNCRNA*), a nucleus-localized lncRNA, enhances drought and salt stress tolerance (Qin et al., 2017). These results suggested that heat stress significantly changed the expression levels of lncRNAs and, thus, affected the function of genes in potato. It also provided additional foundation for further functional characterization of genes.

### 4.3 AS events in responses to heat stress

AS is a critical post-transcriptional regulation influencing plant stress responses. In eukaryotes, it could increase the complexity and adaptability of systems by increasing transcriptional and protein diversity (Lorkovic et al., 2000). Previous studies indicated that greater than 60% of intron-containing genes underwent AS events in Arabidopsis (Marquez et al., 2012). Notably, the data relying on Illumina RNA-seq have confirmed that abiotic stress significantly alters AS in plants (Calixto et al., 2018). However, Illumina RNA sequencing is unable to accurately obtain each isoform due to the short-sequencing reads. SMRT-seq has been performed to analyze AS events in plants, which greatly increased the sensitivity of isoform identification (Xu et al., 2015; Wang et al., 2016). Here, we combined NGS and SMRT sequencing to generate more accurate AS analysis. In total, 22,125 genes underwent AS events. RI, SE, A3, and A5 were markedly induced at different stages, with RI events being the most frequent under heat stress. This is consistent with previous studies in Arabidopsis (Marquez et al., 2012). As previously reported, the heat shock factor *HsfA2* has been shown to produce *HsfA2-II* and *HsfA2-III* splicing isoforms at moderate and high temperatures, respectively (Liu et al., 2013). AS of *TaHSFA6e* modulates HSP-mediated translational regulation in response to heat stress in wheat (Wen et al., 2023). The results indicated that AS was an important post-transcriptional regulatory event under heat stress. According to WGCNA analysis, the correlation between heat stress treatment for 0.5h and AS was the highest. The significantly correlated blue module included genes *RHC06H2G3042*, *RHC09H1G2918*, *RHC02H2G1799*, *RHC11H2G1891*, and *RHC08H1G2153*. RHC06H2G3042 and RHC09H1G2918 encode the peptidylprolyl isomerase, belonging to the FKBP (FK506 binding protein) family. As the homologous gene of *RHC06H2G3042*, ROF1 (FKBP62) was shown to be involved in long term acquired thermotolerance by its interaction with HSP90.1 and modulation of the heat shock TF HsfA2 in Arabidopsis (Meiri and Breiman, 2009). ROF2, a homologous gene of *RHC09H1G2918*, is transcribed by HsfA2, which is also essential for the maintenance of ROF2 during recovery from heat stress. ROF2 was interact with ROF1 and the heterodimers ROF1/ROF2 abrogate HsfA2 transcription activity, which in turn leads to the negative regulation of long-term acquired thermotolerance by ROF2 (Meiri et al., 2010). In Arabidopsis, heat-stress-associated 32-kD protein (Hsa32), as the homologous gene of *RHC02H2G1799*, is essential for acquired thermotolerance during long recovery after acclimation (Charng et al., 2006). As the homologous gene of *RHC11H2G1891*, cytosolic HSC70 represses heat stress tolerance and enhances seed germination under salt stress conditions in Arabidopsis (Zhao et al., 2021). HOP2 (HSP70-HSP90 organizing protein 2), a homologous gene of *RHC08H1G2153*, is a member of cytosolic cochaperones and plays a major role in long-term acquired thermotolerance in Arabidopsis (Fernandez-Bautista et al., 2018). Furthermore, GO analysis showed that the genes were related to protein folding or refolding. Protein stability is highly sensitive to temperature, and protein unfold will aggregate when kept at high temperature (Dave and Gruebele, 2015). Therefore, maintaining the functional conformation of proteins is particularly important for the survival of plants under heat stress (Lee and Vierling, 2000). The above data indicated that AS could increase their protein diversity under heat stress and further improve the ability of heat tolerance in adverse environments.

## 5 Conclusion

**Figure 8:**
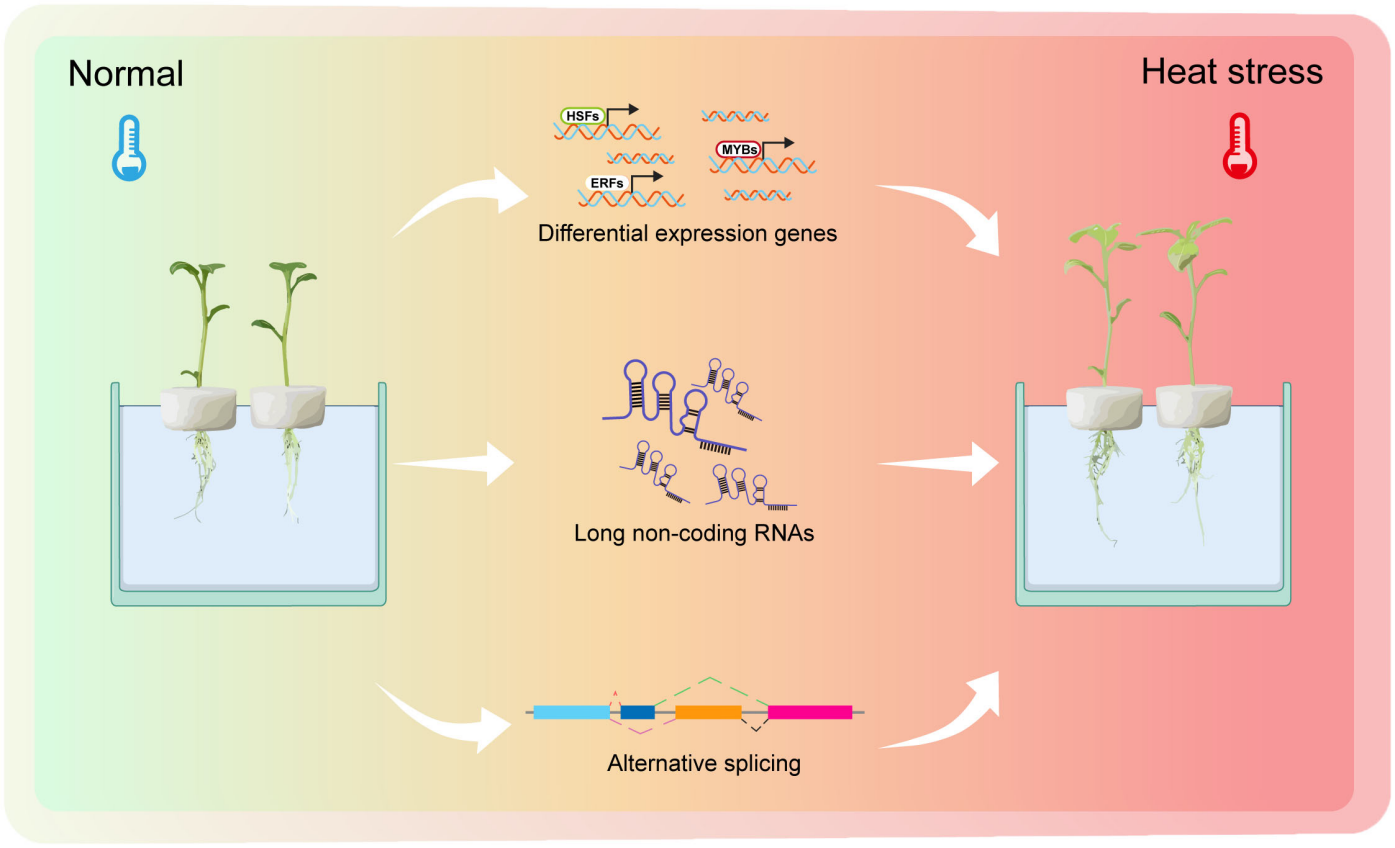
A working model of potato plants response to heat stress. Normal temperature represents 22°C. Heat stress is defined as 35°C.The picture on the left represents potato plants growing under normal conditions with green leaves. The picture on the right represents potato plants experiencing heat stress, with the plant’s leaves slowly turning yellow and with dead spots in the leaves. The whole process of experiencing heat stress is accompanied by many differential expression genes (DEGs), differential expression long non-coding RNAs (DELs) and alternative splicing (AS) events.

In this study, we combined the advantages of NGS and SMRT techniques to make the differential expression genes of potato leaves at different time points under heat stress. Furthermore, we identified 13,159 DEGs, including 950 DETFs. GO enrichment analysis revealed that they were involved in temperature signal, phytohormone, and protein modification. We also found that there were 2,048 putative lncRNAs and 22,125 AS events. These are potential resources for the study of potato tolerance under heat stress (**Figure 8**).

## Data availability statement

The datasets presented in this study can be found in online repositories. The names of the repository/repositories and accession number(s) can be found below: Bioproject accession PRJNA1007236.

## Author contributions

LS: Writing – original draft. YZ: Formal Analysis, Writing – review & editing. SW: Data curation, Writing – review & editing. KW: Software, Writing – review & editing. YL: Data curation, Writing – review & editing. MZ: Data curation, Writing – review & editing. HJ: Supervision, Writing – review & editing. DL: Supervision, Writing – review & editing.

## Supplementary material

Supplementary Figure 1 Phenotype figures of potato after heat stress treatment. **(A**–**F)** Potato plants at different times after heat stress treatment. h, hour. **(G)** Potato plants after 10 days of heat stress treatment. d, day. **(H)** magnified map of red dashed area in G.

Supplementary Figure 2 PCA of the transcriptomes of the 24 samples.

Supplementary Figure 3 Expression profile of HSPs after heat stress treatment.

Supplementary Figure 4 The numbers of differentially expressed genes at different time points after heat stress.

Supplementary Table 1 The primer sequences used for qRT-PCR validation in this study.

Supplementary Table 2 Summary of Illumina transcriptome sequencing data mapping on potato reference genome.

Supplementary Table 3 Statistics of the number of differentially expressed genes.
