# Supplementary figures and images for: Construction of heat stress regulation networks based on Illumina and SMRT sequencing data in potato
Source: Front Plant Sci. 2023 Nov 2;14:1271084. doi: 10.3389/fpls.2023.1271084 (PMC10651764; doi:10.3389/fpls.2023.1271084)

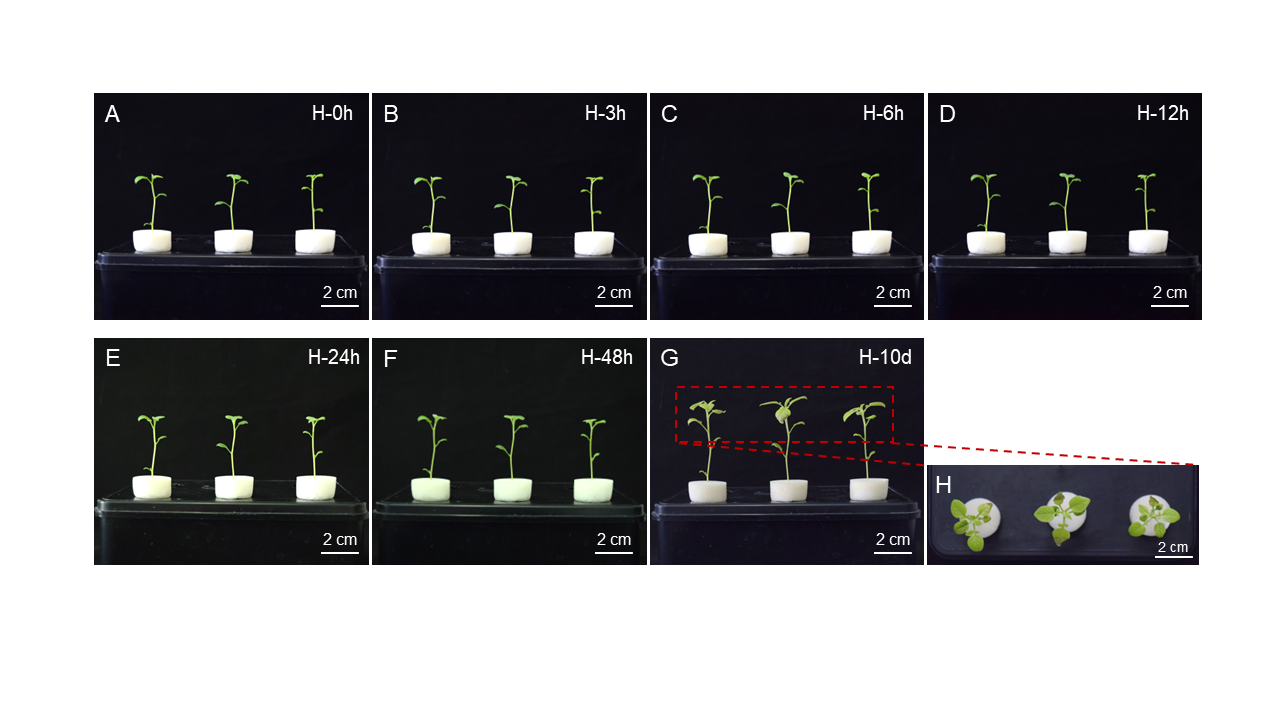

Supplement: Supplementary Figure 1 — Phenotype figures of potato after heat stress treatment. (A–F) Potato plants at different times after heat stress treatment. h, hour. (G) Potato plants after 10 days of heat stress treatment. d, day. (H) magnified map of red dashed area in G. [file Image_1.tif]

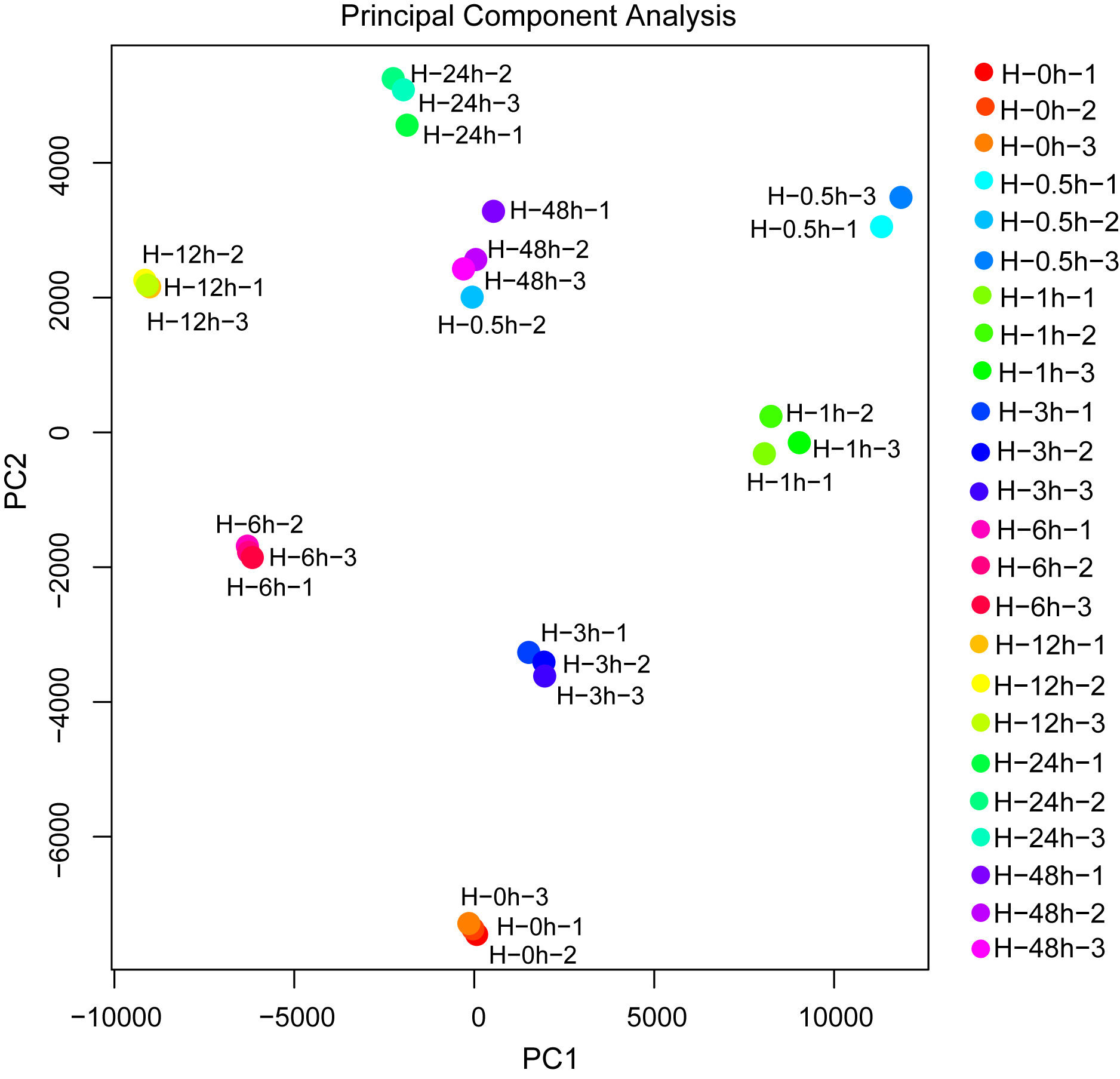

Supplement: Supplementary Figure 2 — PCA of the transcriptomes of the 24 samples. [file Image_2.tif]

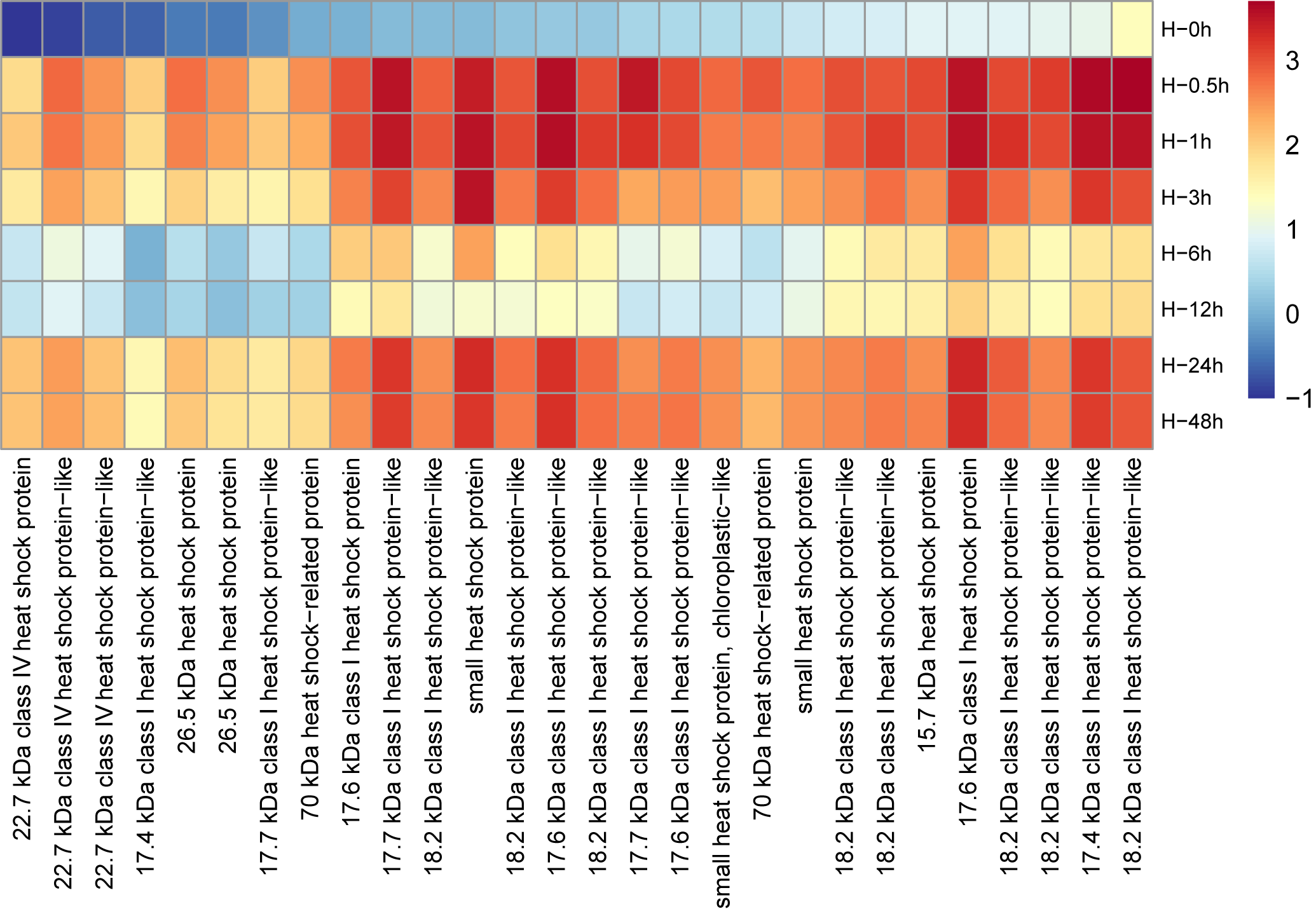

Supplement: Supplementary Figure 3 — Expression profile of HSPs after heat stress treatment. [file Image_3.tif]

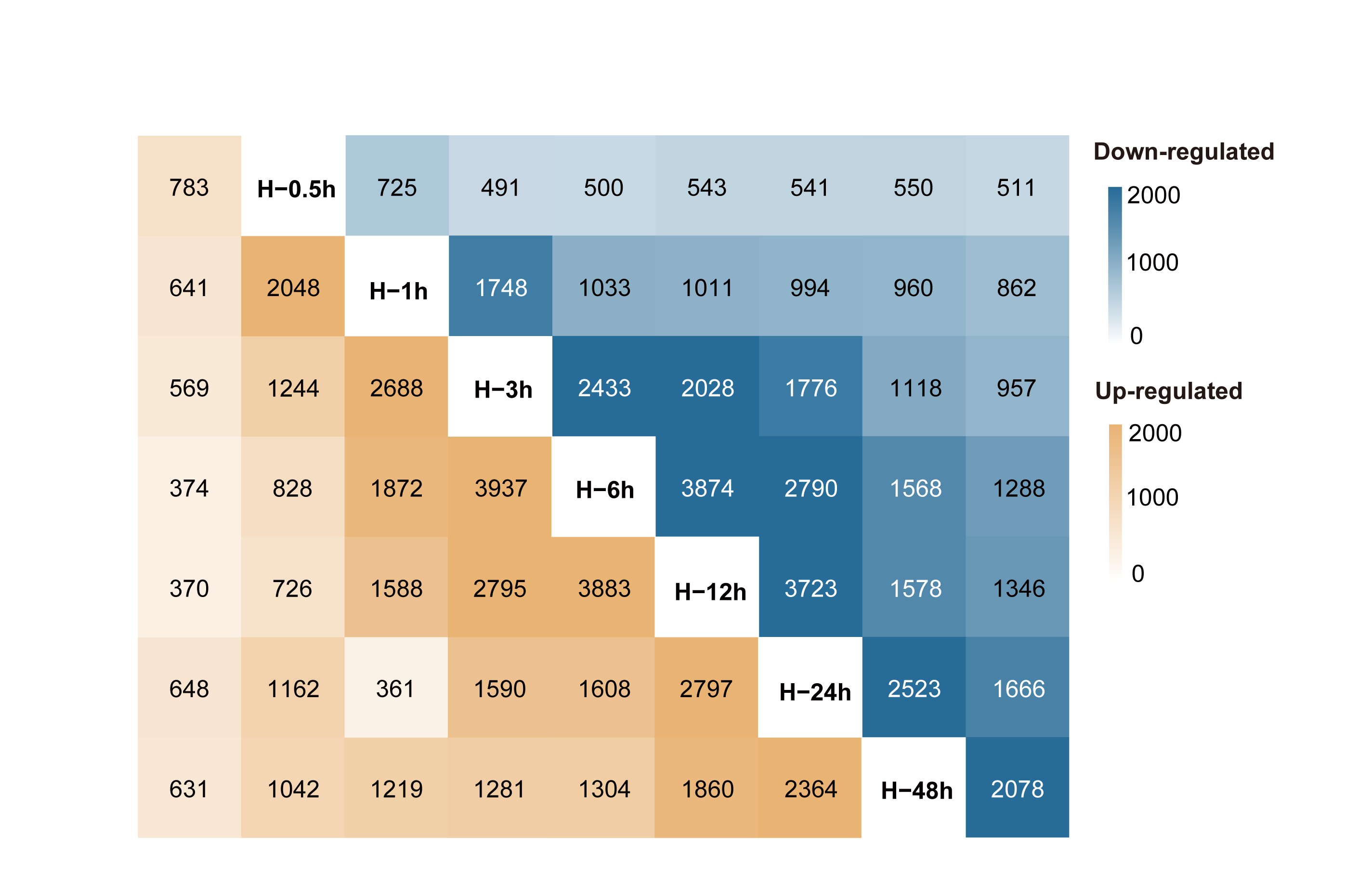

Supplement: Supplementary Figure 4 — The numbers of differentially expressed genes at different time points after heat stress. [file Image_4.tif]
